# Supplementary material for: Shifts in ruminant fermentation during inhibition of methanogenesis are reflected in the isotope compositions of volatile fatty acids
Source: Appl Environ Microbiol. 2026 May 29;92(6):e00489-26. doi: 10.1128/aem.00489-26 (PMC13274383; doi:10.1128/aem.00489-26)
Supplement: Supplemental material — Description of isotopic model and tables of raw data, including saliva buffer composition, concentrations of gases, dissolved volatile fatty acids, and their isotope compositions. [file aem.00489-26-s0001.pdf]

AMERICAN SOCIETY FOR MICROBIOLOGY
Journals.ASM.org

Shifts in ruminant fermentation during inhibition of methanogenesis are reflected in the isotope compositions of volatile fatty acids.

Elliott P. Mueller,[1,2]* Rich Duong,[3] John Eiler,[1] Matthias Hess, [3] Alex Sessions [1]

[1]Division of Geological and Planetary Sciences, California Institute of Technology, Pasadena, CA, USA

[2]Department of Geological Sciences, University of Colorado Boulder, Boulder, CO, USA

[3]College of Agriculture and Environmental Science, University of California Davis, Davis, CA, USA

[*]Address correspondence to Elliott Mueller, elliottpmueller@gmail.com

Present address: Department of Geologicl Sciences, University of Colorado Boulder, Boulder, CO, USA.

Supplemental material

Constraining branching ratios with isotope compositions

In the main text, the carbon isotopic offset between acetate and feed is used as a proxy for the the branching ratio (f_{acc}) of fluxes at the acetyl-CoA node of metabolism, where f_{acc} is the fraction of acetyl-CoA that is used for acetate synthesis (Figure S1). For the purposes of this initial study, it represents an overly simplified description of fermentative metabolism. However, regardless of the nuance of the model parameters or assumptions, the directionality of the proxy remains true: As f_{acc} increases, the positive offset between acetate δ^{13}C and feed δ^{13}C values will increase and vice versa. Thus, even in the absence of this quantitative proxy, the isotopic enrichment of acetate holds valuable information. The following section describes the quantitative proxy mathematically.

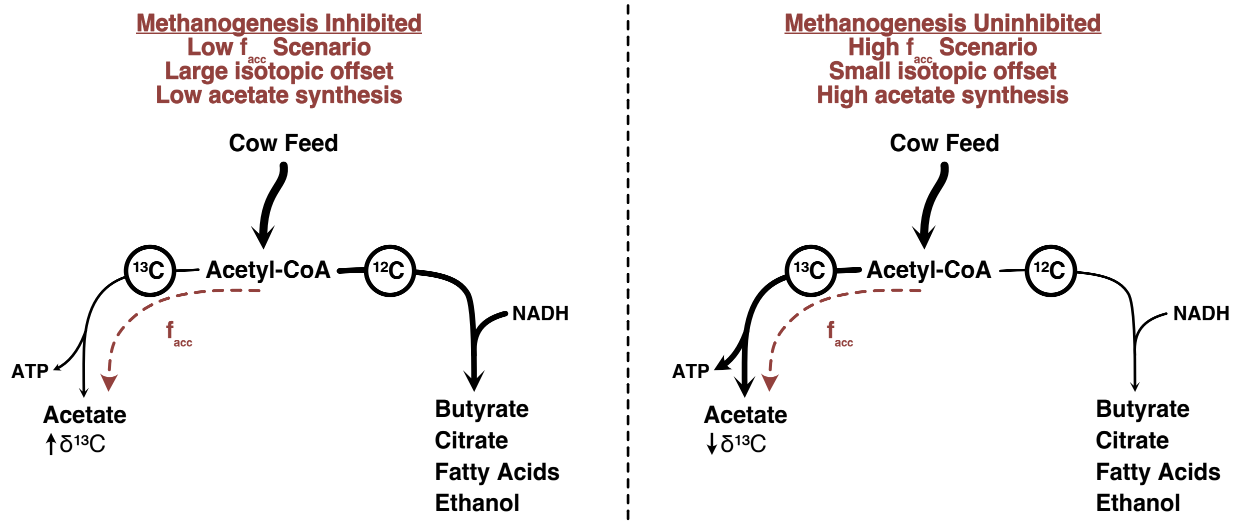

FIG 1 The acetyl-CoA branch point of metabolism constrained by the carbon isotope offset between cow feed and acetate. Negative controls were characterized by low offsets caused by high branching ratios toward acetate synthesis (right). Meanwhile, incubations where methanogenesis was inhibited were characterized by high offsets caused by low branching ratios toward acetate synthesis (left). Adapted from [1]

First we define f_{acc}

$$f_{acc} = \frac{\phi_{acetate}}{\phi_{acetylCoA}} \tag{S1}$$

Where $\phi_{acetate}$ is the flux of acetate synthesis and $\phi_{acetyl-CoA}$ is the flux of acetyl-CoA production from upstream metabolism.

By mass balance, the weighted average isotope composition of all products synthesized from acetyl-CoA should be equivalent to the isotope composition of the feed on average:

$$\delta^{13}C_{acetyl-CoA} = f_{acc}\delta^{13}C_{acetate} + (1 - f_{acc})\delta^{13}C_{other} \tag{S2}$$

Where $\delta^{13}C_{other}$ represents the carbon isotope composition of all metabolic products synthesized from acetyl-CoA other than acetate and $f_{acc} + f_{other} = 1$

It was shown in a previous study of fermentative bacteria that minimal carbon isotope fractionations (∼1‰) occur during the synthesis of acetyl-CoA from a starting sugar substrate, so we assume that synthesized acetyl-CoA has the same isotope composition as the feed ($\delta^{13}C_{OM}$).

$$\delta^{13}C_{OM} = f_{acc}\delta^{13}C_{acetate} + (1 - f_{acc})\delta^{13}C_{other} \tag{S3}$$

The kinetic isotope effect (KIE) for acetate synthesis ($\varepsilon_{acetate}$) and other syntheses (ε_{other}) are expressed on a steady state pool of acetyl-CoA, which is balanced between production upstream from glycolysis and consumption by these downstream reactions. This KIE is a measurable difference in the rate of the reaction between the isotopologues of acetyl-CoA or any substrate. Typically, enzymes have faster reaction rates with the lighter isotope (e.g. ^{12}C, ^{1}H) compared to the heavier isotope (e.g. ^{13}C, ^{2}H) which leaves the substrate enriched in the heavier isotope, leadings to a more positive $\delta^{13}C$ or $\delta^{2}H$ value. In this case, the steady state pool of acetyl-CoA has a distinct isotope composition

($\delta^{13}C_{acetyl_{CoA,ss}}$) from the "instantaneously" synthesized acetyl-CoA, because of these KIEs that leave the acetyl-CoA ^{13}C -enriched. See [2] for more details.

$$\varepsilon_{acetate} = \delta^{13}C_{acetylCoA,ss} - \delta^{13}C_{acetate} \tag{S4}$$

$$\varepsilon_{other} = \delta^{13}C_{acetylCoA,ss} - \delta^{13}C_{other} \tag{S5}$$

We assume that $\varepsilon_{acetate}$ is zero, in line with previous studies [1, 3] as it involves no bond cleavage, condensation, carbon atom hybridization changes or redox changes (i.e. $\delta^{13}C_{acetate} = \delta^{13}C_{acetylCoA,ss}$). Substituting Equations S4 and S5 into Equation S3 and solving for f$_{acc}$then yields:

$$f_{acc} = 1 - \frac{\delta^{13}C_{acetate} - \delta^{13}C_{OM}}{\varepsilon_{other}} \tag{S6}$$

Equation 6 demonstrates that when all of acetyl-CoA is used to make acetate (f$_{acc}$ = 1), the isotopic offset is 0. As f$_{acc}$ decreases, the offset approaches ε_{other}.

The reactions encompassed in ε_{other} include citrate synthase (to the TCA cycle), acetyl-CoA carboxylase (to fatty acid biosynthesis), acetyl-CoA acyltransferase (to butyrate production), and alcohol dehydrogenase (to ethanol production). In previous work these enzymes were found to have similar magnitude KIEs from 5-15‰, and assume for simplicity that ε_{other} is 10‰ in the main text. This assumption allows us to explicitly calculate f$_{acc}$ at a given offset value. For example, the offset increased from 2-3‰ to 6-8‰ between with and without *A. taxiforms* , respectively. Calculated f$_{acc}$ change from 0.7-0.8 and 0.2-0.4 between controls and positive treatments (Figure S1). This indicates that the branching ratio toward acetate synthesis at the acetyl-CoA node of metabolism decreased by 30-50% when methanogenesis was added, consistent with a 42-66% drop in acetate synthesis and up to 30% increase in butyrate synthesis across the various feed types (Figure 3, main text).

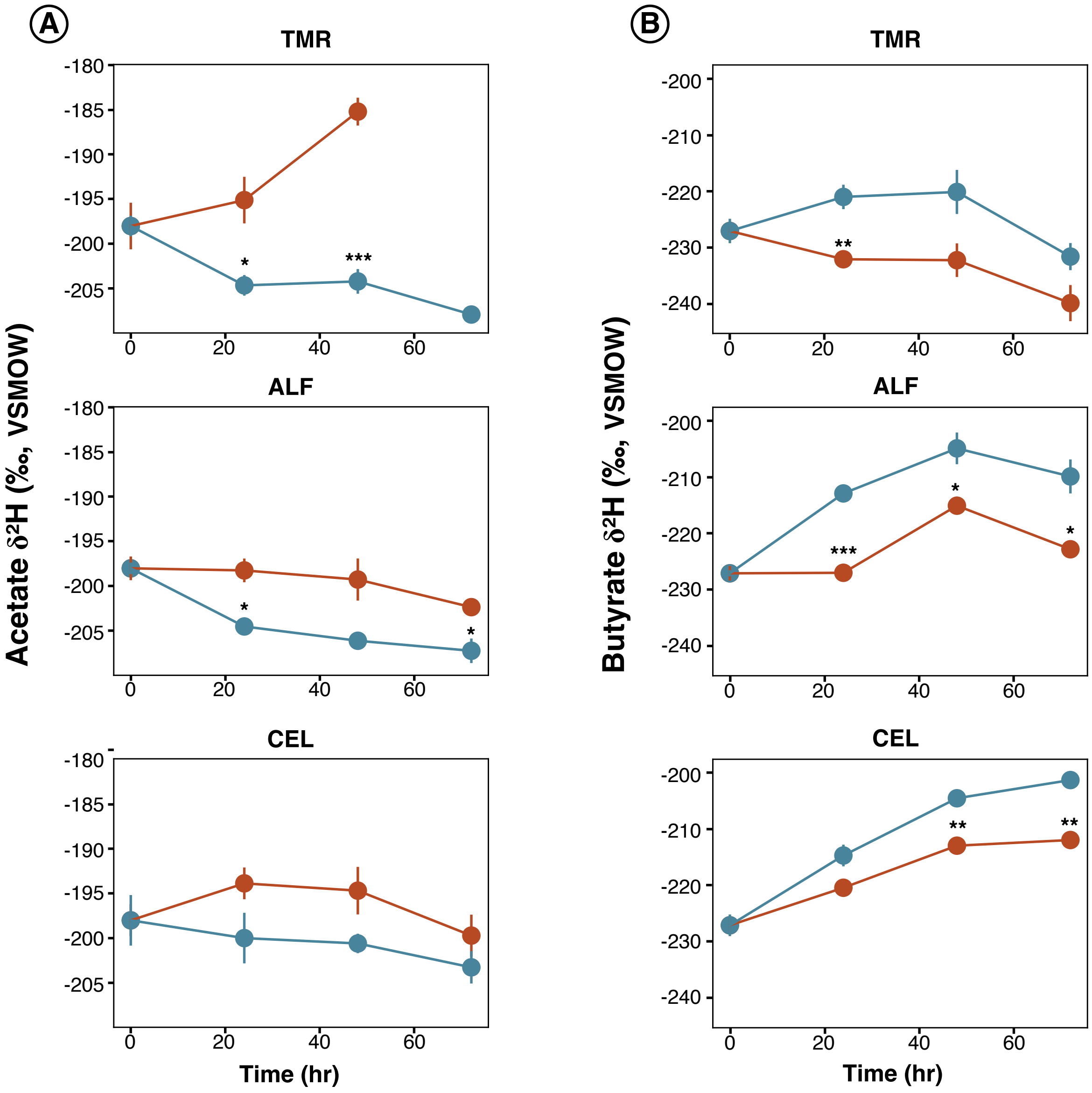

FIG 2 Hydrogen isotope composition of acetate and butyrate with time. The two VFAs show opposite signals upon addition of *A. taxiforms* . This suggests that NADH and water did not systematically change isotope composition, since we would expect the shifts in $\delta^2 H$ of acetate and butyrate to covary in that case. Error bars represent standard error on the mean of four incubation replicates. (*P<0.05, **P<0.01, ***P<0.001)

TABLE 1 HESI-Orbitrap MS Parameters

**ESI Parameters**	
Polarity	Negative
Sheath Gas Flow	10
Auxiliary Gas Flow	3
Sweep Gas Flow	3
Spray Voltage	3.1
Spray Current	<0.2 μA
Auxiliary Gas Temperature	100°C
Capillary Temperature	320°C
**Acetate MS Parameters**	
Time	0 — 11 minutes
Quadrupole Filter Range	57 - 62 m/z
Resolution	60,000 (at 200 m/z)
AGC	1×10^6
Microscans	1
S-Lens Radio Frequency Level	60%
**Propionate MS Parameters**	
Time	11 — 17 minutes
Quadrupole Filter Range	72 - 75 m/z
Resolution	60,000 (at 200 m/z)
AGC	1×10^6
Microscans	1
S-Lens Radio Frequency Level	60%
**Butyrate MS Parameters**	
Time	17 — 24 minutes
Quadrupole Filter Range	85 - 90 m/z
Resolution	120,000 (at 200 m/z)
AGC	1×10^6
Microscans	1
S-Lens Radio Frequency Level	60%

TABLE 2 Randomization of Ankom vessels and their endpoint pH measured after 72 hours. Positive and negative treatements indicate incubations with and

without *A. taxiforms*, respectively. Abbreviations: CEL, cellulose; TMR, total mean ration; ALF, alfalfa

**Vessel #**	**Feed Type**	**Treatment**	**Endpoint pH**
1	ALF	Positive	5.90
2	ALF	Negative	5.74
3	TMR	Negative	5.26
4	ALF	Negative	5.67
5	CEL	Negative	5.99
6	TMR	Positive	5.95
7	ALF	Negative	5.65
8	ALF	Positive	5.93
9	CEL	Negative	6.06
10	CEL	Negative	5.87
11	CEL	Positive	6.27
12	TMR	Positive	5.53
13	ALF	Negative	5.65
14	CEL	Negative	5.87
15	TMR	Positive	5.43
16	CEL	Positive	6.25
17	CEL	Positive	6.26
18	TMR	Negative	5.23
19	ALF	Positive	6.02
20	TMR	Negative	5.33
21	TMR	Negative	5.10
22	CEL	Positive	6.25
23	TMR	Positive	6.49
24	ALF	Positive	5.94

TABLE 3 Gas production at 24 hour timepoint.

Vessel #	Cond	Total Gas (mL)	CH_4 (mL/g)	CO_2 (mL/g)	CH_4 (‰)	CO_2 (‰)
1	ALF+	210	0.3	36.1	-57.9	-14.8
2	ALF-	168	9.7	14.5	-59.7	-11.9
3	TMR-	200	11.2	17.9	-58.3	-10.3
4	ALF-	180	12.0	17.8	-61.6	-12.2
5	CEL-	122	5.3	9.6	-56.0	-8.6
6	TMR+	160	0.2	18.2	-59.4	-14.2
7	ALF-	240	16.9	31.5	-62.5	-12.6
8	ALF+	172	0.5	25.7	-55.7	-15.1
9	CEL-	126	6.6	12.9	-60.7	-9.8
10	CEL-	150	10.0	14.7	-59.3	-9.2
11	CEL+	105	0.9	8.6	-54.6	-12.4
12	TMR+	240	0.6	43.5	-55.8	-14.8
13	ALF-	210	13.8	22.7	-60.7	-12.3
14	CEL-	131	10.6	15.5	-59.4	-9.2
15	TMR+	192	0.4	52.2	-56.4	-13.8
16	CEL+	128	1.1	17.5	-53.3	-13.2
17	CEL+	127	1.2	13.0	-56.9	-13.1
18	TMR-	196	12.7	24.6	-58.8	-10.9
19	ALF+	158	0.5	22.1	-58.4	-15.2
20	TMR-	182	11.0	20.1	-58.1	-10.8
21	TMR-	205	15.5	37.3	-58.5	-10.6
22	CEL+	131	1.0	21.2	-51.5	-12.1
23	TMR+	205	0.4	34.4	-53.2	-13.2
24	ALF+	148	0.3	21.9	-58.1	-13.9

TABLE 4 Gas production at 48 hour timepoint.

Vessel #	Cond	Total Gas (mL)	CH_4 (mL/g)	CO_2 (mL/g)	CO_2 (‰)	CH_4 (‰)
1	ALF+	161	0.0	29.5	-18.4	—
2	ALF-	182	7.7	25.1	-14.5	-60.8
3	TMR-	175	9.3	29.6	-13.0	-60.6
4	ALF-	186	7.5	27.6	-15.2	-63.3
5	CEL-	96	1.8	12.9	-10.0	-65.5
6	TMR+	187	0.0	36.2	-17.5	—
7	ALF-	190	9.0	37.9	-15.8	-62.4
8	ALF+	151	0.0	28.58	-17.8	—
9	CEL-	92	1.2	8.9	-9.4	-61.5
10	CEL-	91	1.6	12.0	-9.3	-63.1
11	CEL+	85	0.0	9.7	-13.2	—
12	TMR+	210	0.0	49.8	-19.2	—
13	ALF-	185	8.5	34.0	-15.1	-62.7
14	CEL-	110	2.2	14.1	-10.3	-64.2
15	TMR+	182	0.0	69.2	-17.7	—
16	CEL+	66	0.0	7.2	-13.5	—
17	CEL+	76	0.0	8.8	-13.4	—
18	TMR-	180	8.7	33.4	-13.5	-59.0
19	ALF+	168	0.0	38.3	-18.6	—
20	TMR-	209	8.5	36.6	-12.2	-57.5
21	TMR-	169	8.5	38.9	-13.4	-58.9
22	CEL+	80	0.0	11.0	-14.0	—
23	TMR+	182	0.0	48.4	-18.0	—
24	ALF+	157	0.1	34.8	-18.6	—

TABLE 5 Gas production at 72 hour timepoint.

Vessel #	Cond	Total Gas (mL)	CH_4 (mL/g)	CO_2 (mL/g)	CO_2 (‰)	CH_4 (‰)
1	ALF+	111	0.0	11.0	-19.6	—
2	ALF-	134	3.8	16.2	-16.2	—
3	TMR-	64	0.6	4.8	-14.8	—
4	ALF-	132	4.2	20.1	-16.8	-64.9
5	CEL-	41	0.1	1.1	-7.1	—
6	TMR+	136	0.0	22.9	-21.0	—
7	ALF-	125	3.5	15.1	-17.4	-70.4
8	ALF+	131	0.0	16.8	-20.7	—
9	CEL-	51	0.5	2.5	-9.5	-65.3
10	CEL-	49	0.3	2.5	-9.1	-63.4
11	CEL+	41	0.0	1.9	-13.1	—
12	TMR+	136	0.0	20.0	-22.1	—
13	ALF-	139	4.6	18.3	-17.2	-69.8
14	CEL-	52	0.5	3.3	-9.8	-64.2
15	TMR+	136	0.0	19.8	-21.5	—
16	CEL+	45	0.0	2.5	-14.4	—
17	CEL+	47	0.0	2.3	-14.3	—
18	TMR-	103	1.8	12.9	-17.2	-59.0
19	ALF+	109	0.0	17.8	-20.5	—
20	TMR-	105	2.0	14.5	-16.4	-55.5
21	TMR-	102	2.0	13.3	-16.2	-55.4
22	CEL+	42	0.0	2.2	-14.2	—
23	TMR+	128	0.0	19.3	-21.4	—
24	ALF+	116	0.0	22.1	-20.5	—

TABLE 6 VFA $\delta^{13}C$ and $\delta^{2}H$ values at 24 hour timepoint.

		$\delta^{13}C$ (‰, VPDB)			$\delta^{2}H$ (‰, VSMOW)		
**Vessel #**	**Cond**	**Ac**	**Pro**	**But**	**Ac**	**Pro**	**But**
1	ALF+	-20.7	-28.1	-32.1	-197	-231	-225
2	ALF-	-23.0	-26.4	-28.9	-203	-214	-212
3	TMR-	-22.2	-25.6	-30.1	-208	-217	-226
4	ALF-	-23.9	-26.9	-30.8	-203	-214	-214
5	CEL-	-21.6	-26.2	-28.6	-195	-214	-217
6	TMR+	-20.7	-28.4	-31.1	-192	-224	-235
7	ALF-	-23.7	-24.9	-29.3	-206	-212	-211
8	ALF+	-21.0	-27.0	-30.6	-198	-219	-231
9	CEL-	-22.4	-25.3	-29.3	-209	-201	-215
10	CEL-	-22.1	-26.5	-27.8	-200	-216	-208
11	CEL+	-19.4	-26.8	-28.3	-197	-224	-218
12	TMR+	-18.7	-27.8	-30.6	-196	-228	-231
13	ALF-	-24.3	-25.0	-28.8	-203	-207	-214
14	CEL-	-22.4	-26.0	-29.1	-195	-201	-219
15	TMR+	-19.7	-26.9	-28.9	-190	-233	-232
16	CEL+	-19.3	-27.7	-28.4	-188	-227	-222
17	CEL+	-20.2	-27.7	-28.3	-194	-226	-224
18	TMR-	-23.4	-25.7	-31.1	-205	-209	-215
19	ALF+	-20.2	-26.5	-29.5	-203	-217	-226
20	TMR-	-22.5	-24.9	-28.7	-201	-213	-220
21	TMR-	-23.2	-25.1	-30.1	-205	-202	-223
22	CEL+	-18.9	-26.1	-28.7	-197	-215	-217
23	TMR+	-19.7	-25.0	-28.2	-203	-215	-230
24	ALF+	-21.2	-26.8	-30.5	-196	-224	-225

TABLE 7 VFA $\delta^{13}C$ and $\delta^{2}H$ values at 48 hour timepoint.

		$\delta^{13}C$ (‰, VPDB)			$\delta^{2}H$ (‰, VSMOW)		
**Vessel #**	**Cond**	**Ac**	**Pro**	**But**	**Ac**	**Pro**	**But**
1	ALF+	-22.0	-26.8	-31.5	-204	-233	-224
2	ALF-	-25.1	-26.1	-28.3	-207	-214	-211
3	TMR-	-22.1	-25.8	-30.0	-206	-212	-227
4	ALF-	-24.4	-26.5	-29.4	-209	-210	-213
5	CEL-	-23.2	-25.3	-27.3	-199	-210	-203
6	TMR+	-19.1	-26.3	-29.1	-185	-233	-225
7	ALF-	-26.9	-27.1	-29.5	-207	-212	-199
8	ALF+	-23.2	-28.5	-29.1	-195	-227	-211
9	CEL-	-24.6	-27.2	-27.8	-200	-200	-208
10	CEL-	-25.1	-27.2	-26.6	-197	-200	-200
11	CEL+	-23.3	-28.0	-28.6	-187	-222	-214
12	TMR+	-19.6	-26.9	-28.5	-181	-221	-228
13	ALF-	-28.3	-27.3	-29.7	-202	-201	-200
14	CEL-	-25.3	-25.5	-28.0	-203	-198	-205
15	TMR+	-17.9	-26.4	-28.6	-186	-229	-239
16	CEL+	-20.6	-26.2	-28.4	-201	-220	-212
17	CEL+	-19.4	-26.4	-28.9	-198	-222	-213
18	TMR-	-23.73	-26.5	-30.0	-202	-212	-226
19	ALF+	-20.7	-27.4	-29.4	-205	-226	-220
20	TMR-	-24.1	-24.5	-30.0	-208	-205	-207
21	TMR-	-25.6	-24.0	-29.3	-201	-219	-221
22	CEL+	-17.9	-28.3	-27.5	-194	-218	-208
23	TMR+	-18.4	-26.4	-28.6	-188	-248	-236
24	ALF+	-23.4	-26.6	-31.3	-200	-215	-214

TABLE 8 VFA $\delta^{13}C$ and $\delta^{2}H$ values at 72 hour timepoint.

		$\delta^{13}C$ (‰, VPDB)			$\delta^{2}H$ (‰, VSMOW)		
**Vessel #**	**Cond**	**Ac**	**Pro**	**But**	**Ac**	**Pro**	**But**
1	ALF+	-21.0	-27.8	-30.2	-201	-251	-227
2	ALF-	-25.7	-27.7	-30.0	-206	-207	-209
3	TMR-	-21.6	-24.6	-30.5	-208	-216	-239
4	ALF-	-25.9	-27.4	-31.7	-204	-206	-211
5	CEL-	-23.2	-26.7	-28.7	-205	-211	-203
6	TMR+	-11.3	-23.7	-29.0	-174	-240	-251
7	ALF-	-26.4	-26.7	-31.4	-208	-213	-218
8	ALF+	-20.1	-27.5	-31.5	-202	-227	-221
9	CEL-	-22.8	-26.5	-29.4	-203	-206	-205
10	CEL-	-23.1	-25.8	-28.3	-208	-215	-197
11	CEL+	-20.6	-25.9	-28.4	-200	-224	-210
12	TMR+	-12.5	-24.9	-29.7	-188	-239	-234
13	ALF-	-25.0	-27.8	-32.4	-211	-215	-201
14	CEL-	-22.4	-25.9	-29.1	-198	-203	-200
15	TMR+	-11.7	-25.6	-29.3	-191	-243	-239
16	CEL+	-19.2	-27.0	-27.1	-193	-223	-210
17	CEL+	-19.0	-26.8	-27.9	-201	-224	-214
18	TMR-	-21.4	-26.6	-30.8	-208	-223	-230
19	ALF+	-22.3	-28.1	-33.1	-204	-243	-223
20	TMR-	-20.7	-25.0	-29.1	-208	-207	-229
21	TMR-	-18.4	-25.2	-28.6	-208	-216	-227
22	CEL+	-18.2	-27.6	-26.4	-206	-219	-213
23	TMR+	-13.4	-24.1	-30.0	-186	-245	-236
24	ALF+	-20.9	-27.3	-30.8	-203	-240	-220

79

TABLE 9 Acetate isotope composition one way ANOVA p-values between positive and negative treatments

Acetate δ^{13}C (‰, VSMOW)			
**Time (hr)**	**ALF**	**CEL**	**TMR**
24	0.0002	0.0002	0.006
48	0.0095	0.01	0.0006
72	0.0001	0.0005	—
**Acetate δ^{2}H (‰, VSMOW)**			
**Time (hr)**	**ALF**	**CEL**	**TMR**
24	0.0103	0.1633	0.02740
48	0.0585	0.1254	0.0002
72	0.0306	0.3385	—

TABLE 10 Propionate isotope composition one way ANOVA p-values between positive and negative treatments

Propionate δ^{13}C (‰, VSMOW)			
**Time (hr)**	**ALF**	**CEL**	**TMR**
24	0.0721	0.0661	0.0631
48	0.1215	0.2623	0.0611
72	0.4273	0.1904	0.2294
**Propionate δ^{2}H (‰, VSMOW)**			
**Time (hr)**	**ALF**	**CEL**	**TMR**
24	0.0199	0.0234	0.0230
48	0.0020	0.0004	0.0152
72	0.0014	0.0032	0.0003

TABLE 11 Butyrate isotope composition one way ANOVA p-values between positive and negative treatments

Butyrate $\delta^{13}C$ (‰, VSMOW)			
**Time (hr)**	**ALF**	**CEL**	**TMR**
24	0.118	0.439	0.755
48	0.245	0.070	0.003
72	0.972	0.028	0.715
**Butyrate $\delta^{2}H$ (‰, VSMOW)**			
**Time (hr)**	**ALF**	**CEL**	**TMR**
24	0.0001	0.0857	0.0068
48	0.0330	0.0071	0.0770
72	0.0148	0.0022	0.1251

TABLE 12 Chemical composition of the saliva buffer added in a 3:1 ratio with rumen fluid to the incubations.

Compound	Concentration (g/L)
$NaCl$	1.09
KCl	0.38
$CaCl_2 \cdot 2H_2O$	0.02
$MgCl_2 \cdot 6H_2O$	0.09
$NaH_2PO_4 \cdot H_2O$	0.46
$Na_2HPO_4 \cdot 12H_2O$	1.19
NH_4Cl	0.09
$NaHCO_3$	2.74

References

[1] E. P. Mueller, V. B. Heuer, J. R. Leadbetter, K.-U. Hinrichs, A. L. Sessions, Metabolic controls on the carbon isotope fractionations of bacterial fermentation, Proceedings of

the National Academy of Sciences 122 (41) (2025) e2511040122. arXiv:https://www.pnas.org/doi/pdf/10.1073/pnas.2511040122, doi:10.1073/pnas.2511040122. URL https://www.pnas.org/doi/abs/10.1073/pnas.2511040122

[2] J. M. Hayes, Fractionation of the Isotopes of Carbon and Hydrogen in Biosynthetic Processes, National Meeting of the Geological Society of America (2001).

[3] H. Penning, R. Conrad, Carbon isotope effects associated with mixed-acid fermentation of saccharides by *Clostridium papyrosolvens*, Geochimica et Cosmochimica Acta 70 (9) (2006) 2283–2297. doi:10.1016/j.gca.2006.01.017.
