# Supplementary figures and images for: Shifts in ruminant fermentation during inhibition of methanogenesis are reflected in the isotope compositions of volatile fatty acids
Source: Appl Environ Microbiol. 2026 May 29;92(6):e00489-26. doi: 10.1128/aem.00489-26 (PMC13274383; doi:10.1128/aem.00489-26)

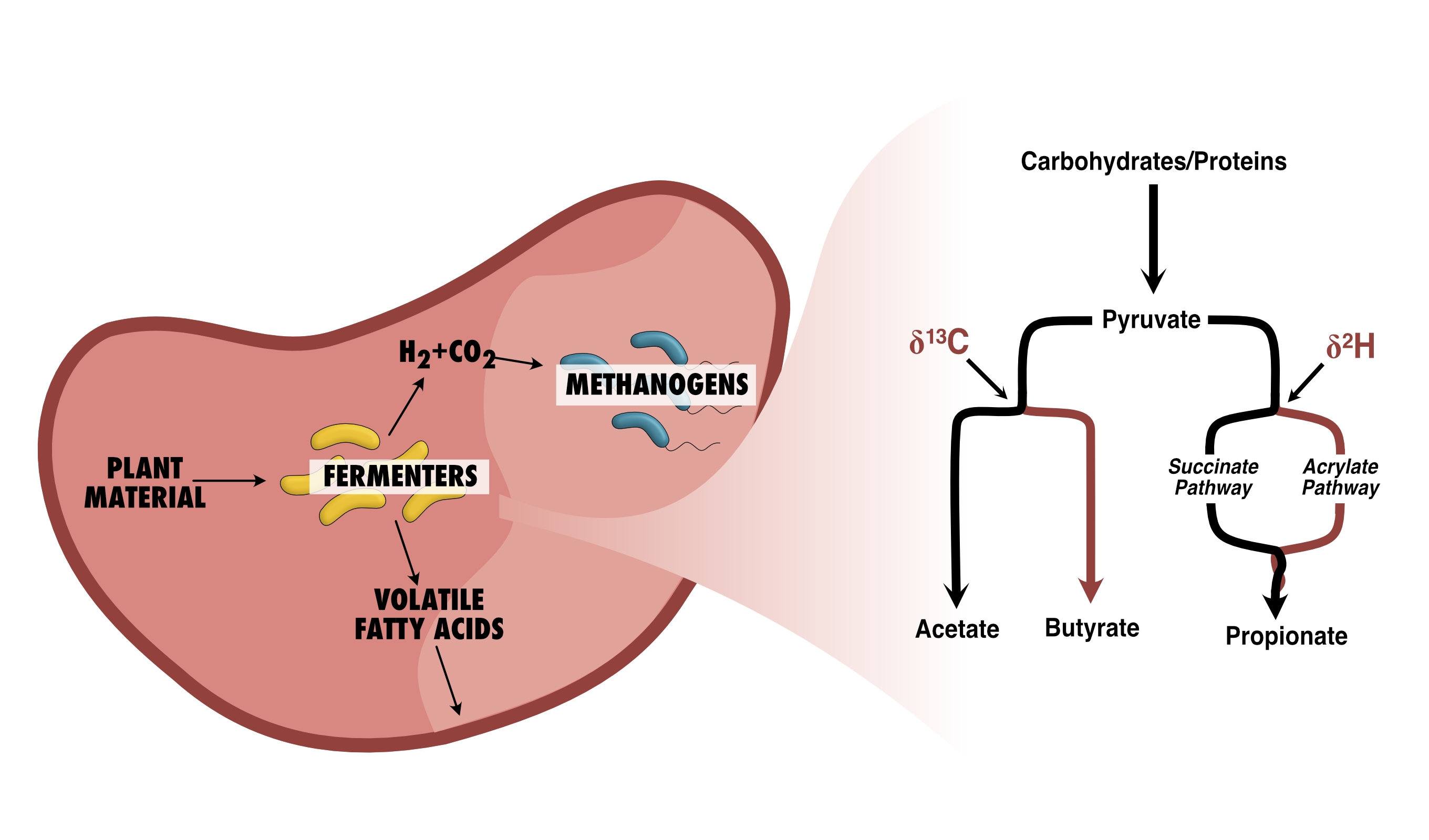

Supplement: Graphical abstract — Visual depiction of the study. [file aem.00489-26-s0002.tiff]
